# Supplementary material for: A Bitter Taste Receptor as a Novel Molecular Target on Cancer-Associated Fibroblasts in Pancreatic Ductal Adenocarcinoma
Source: Pharmaceuticals (Basel). 2023 Mar 3;16(3):389. doi: 10.3390/ph16030389 (PMC10058050; doi:10.3390/ph16030389)
Supplement: Supplementary file 1 [file pharmaceuticals-16-00389-s001.zip › pharmaceuticals-2107205 Supplemental-Figures.pdf]

pharmaceuticals

MDPI

Supplemental Material

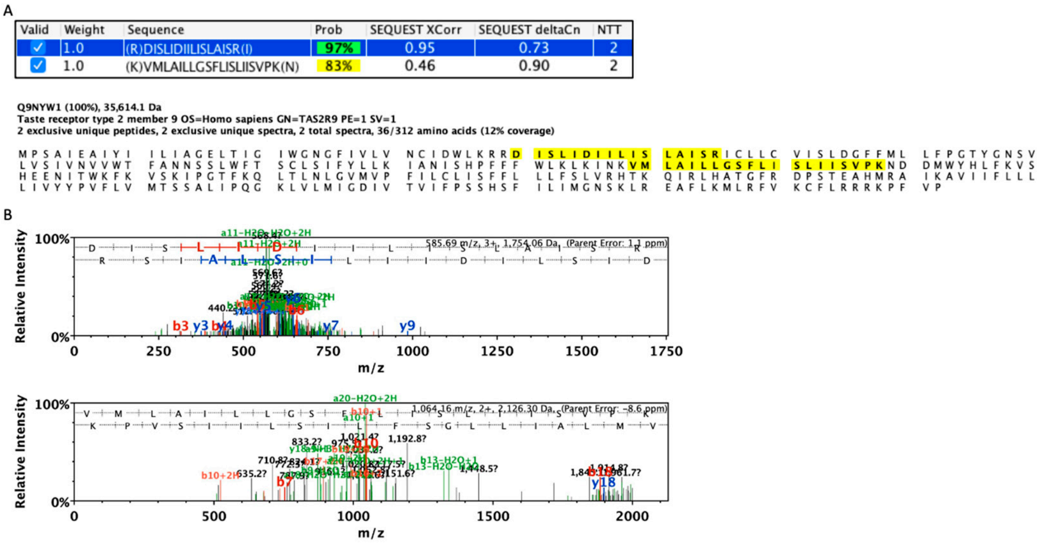

Figure S1. HTTIPKV phage pulldown. (A) Sequences of the 2 identified peptides following MS/MS analysis of the gel band derived from the HTTIPKV phage pulldown assay. Sequences (highlighted) were overlaid on the amino acid sequence of human taste 2 receptor member 9. The percent coverage (MS/MS peptides/TAS2R9 amino acid sequence is 12%. (B) The spectra for each of the 2 identified peptides.

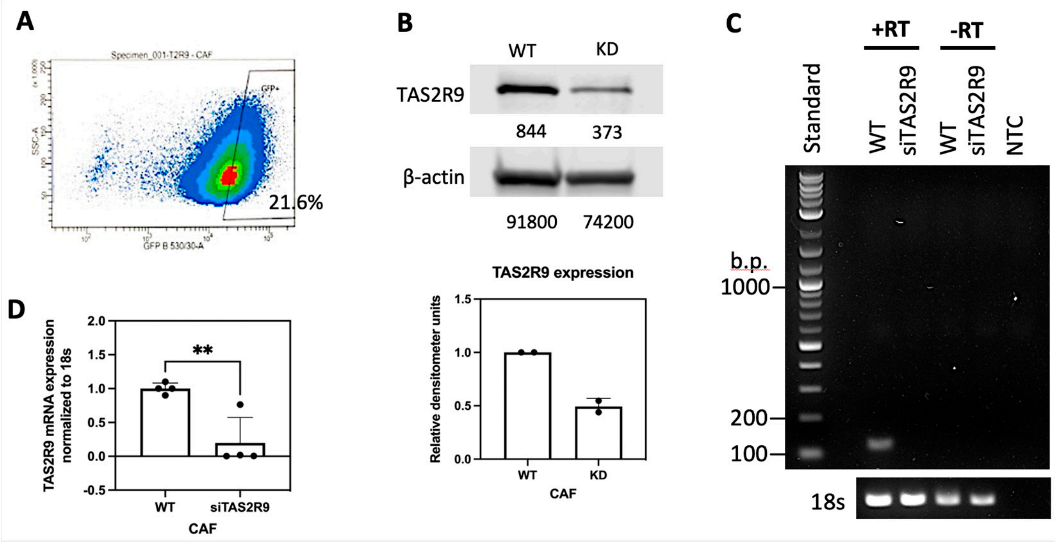

Figure S2. TAS2R9 qPCR Probe Validation. (A) TAS2R9 siRNA lentivirus transduced CAF was selected based on the GFP expression using the Aria Cell Sorter. (B) Immunoblot detection of TAS2R9 expression in the WT and KD CAFs. β-actin serves as a loading control. Densitometry of the TAS2R9 WB image, N = 2. (C) End-point qPCR samples of wildtype (WT) and TAS2R9 siRNA KD reactions were resolved on a 1.5% agarose gel and imaged for SYBR safe intensity. Gel represents reaction samples following TAS2R9 and 18s primer set amplification, respectively. RT = reverse transcriptase; NTC = no template control. (D) Quantification of the qPCR analysis of the TAS2R9 transcriptional levels normalized to 18s. **p=0.0053, N = 4.

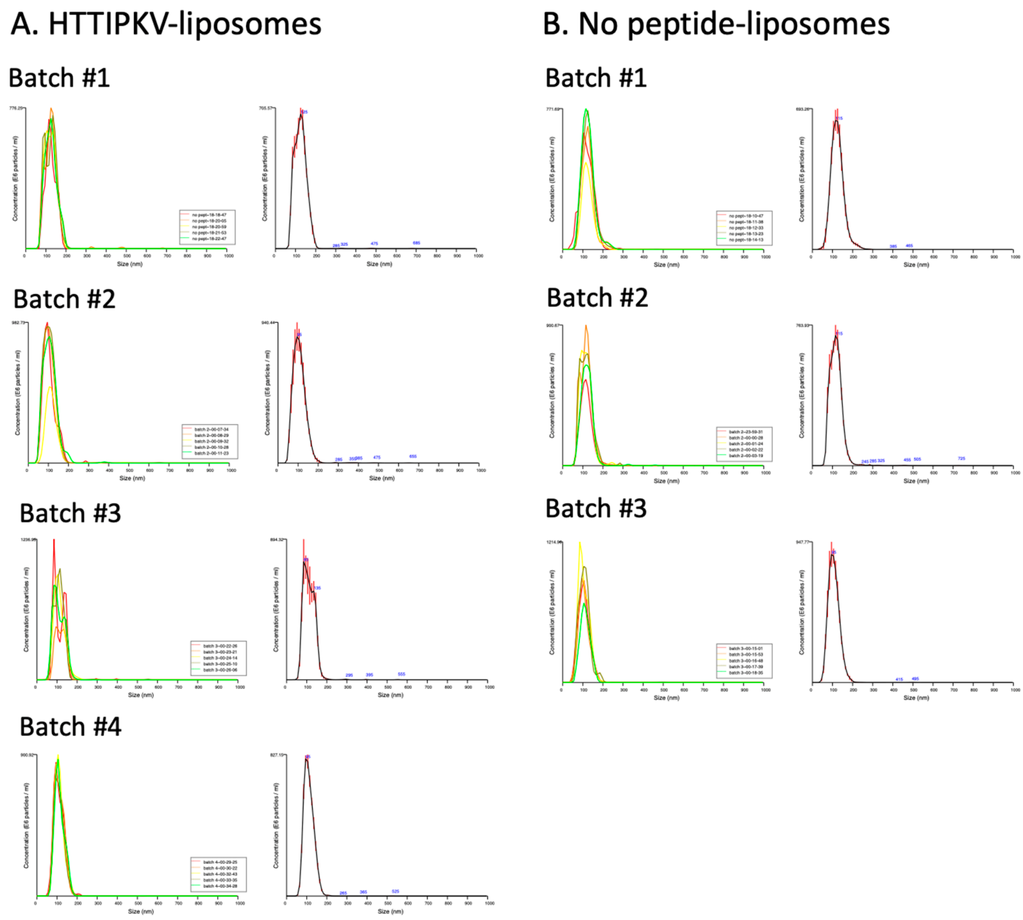

Figure S3. Liposome size distribution determined by NanoSight. (A) HTTIPKV liposomes batch #1-4. (B) No peptide liposomes batch #1-3.

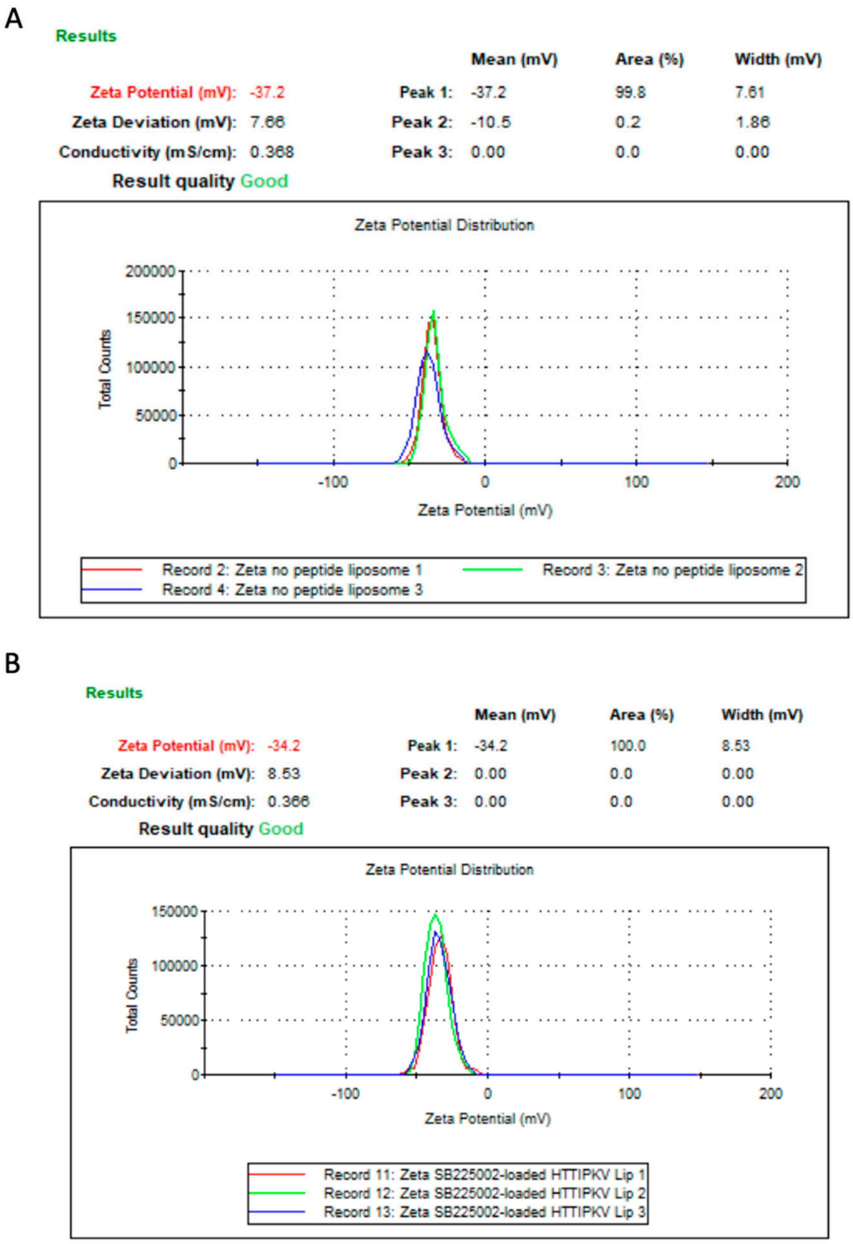

Figure S4. Liposome zeta-potential determined by Zetasizer. (A) No peptide liposome. (B) HTTIPKV liposome.

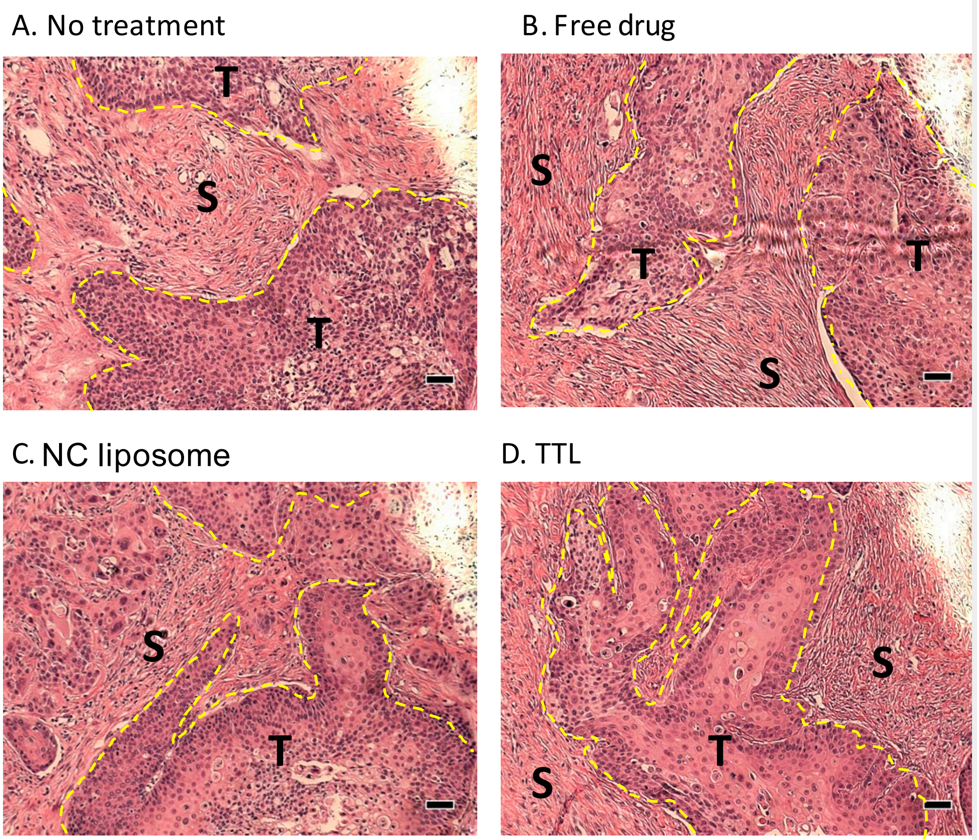

Figure S5. H&E staining of admix BXPC3/CAF tumors given (A) no treatment, (B) free drug, SB225002, (C) SB225002-loaded no-peptide liposomes, and (D) SB225002-loaded HTTIPKV liposomes. Scale bars, 50 μm.
